# Anti-schistosomal action of the calcium channel agonist FPL-64176

**DOI:** 10.1016/j.ijpddr.2019.08.006

**Published:** 2019-09-14

**Authors:** Paul McCusker, John D. Chan

**Affiliations:** Department of Cell Biology, Neurobiology & Anatomy, Medical College of Wisconsin, Milwaukee, WI, 53226, USA

**Keywords:** Schistosomiasis, FPL-64176, Calcium, Anthelmintic, Tegument

## Abstract

Subversion of parasite neuromuscular function is a key strategy for anthelmintic drug development. Schistosome Ca^2+^ signaling has been an area of particular interest for decades, with a specific focus on L-type voltage-gated Ca^2+^ channels (Ca_v_s). However, the study of these channels has been technically challenging. One barrier is the lack of pharmacological probes that are active on flatworms, since the dihydropyridine (DHP) based ligands typically used to study Ca_v_s are relatively ineffective on schistosomes. Here, we have characterized the effect of a structurally distinct putative L-type Ca_v_ agonist, FPL-64176, on schistosomes cultured *ex vivo* and in an *in vivo* murine model of infection. Unlike DHPs, FPL-64176 evokes rapid and sustained contractile paralysis of adult *Schistosoma mansoni* reminiscent of the anthelmintic praziquantel. This is accompanied by tegument disruption and an arrest of mitotic activity in somatic stem cells and germ line tissues. Interestingly, this strong *ex vivo* phenotype was temperature dependent, with FPL-64176 treatment being less potent at 37 °C than 23 °C. However, FPL-64176 caused intra-tegument lesions at the basement membrane of worms cultured *ex vivo* under both conditions, as well as an *in vivo* hepatic shift of parasites from the mesenteric vasculature of infected mice to the liver. Gene expression profiling of worms harvested following *in vivo* FPL-64176 exposure reveals differences in transcripts associated with muscle and extracellular matrix function, as well as female reproduction, which is consistent with the worm phenotypes observed following *ex vivo* drug treatment. These data advance FPL-64176 as a useful tool to study schistosome Ca^2+^ signaling, and the benzoyl pyrrole core as a hit compound that may be optimized to develop new parasite-selective leads.

## Highlights

• The non-dihydropyridine voltage-gated Ca^2+^ channel agonist FPL-64176 paralyzes schistosomes.
• FPL-64176 causes extensive vacuolization near the tegument basement membrane.
• FPL-64176 shifts worms to the liver *in vivo* and alters expression of muscle and germ line transcripts.

## Graphical abstract

**Figure:**
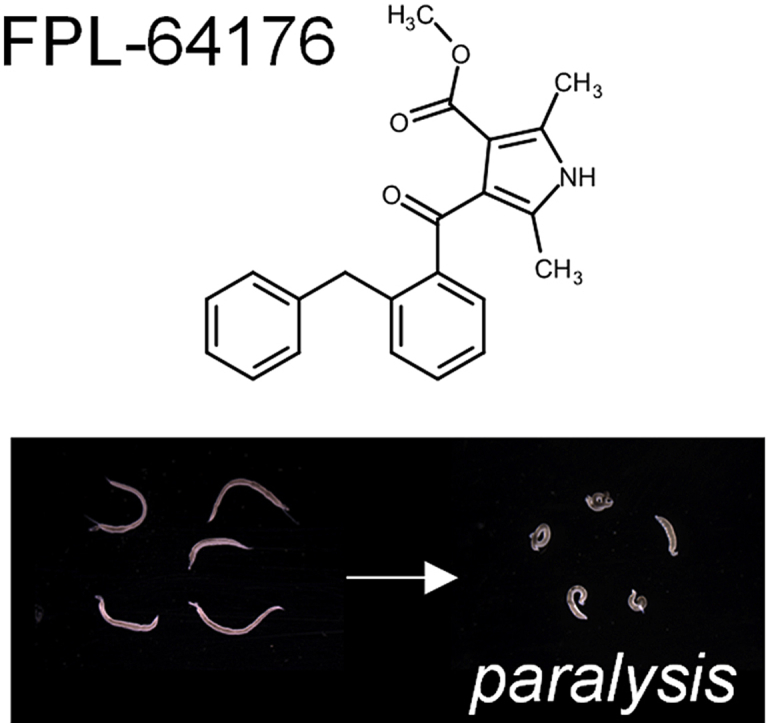

## 1 Introduction

Over 200 million people worldwide are infected with the worms that cause the neglected tropical disease schistosomiasis. These parasites are treated by just one clinical therapy, praziquantel (PZQ) (Hotez and Fenwick, 2009). Arguably, the lack of pharmacological therapies targeting these parasitic worms is due to our incomplete understanding of schistosome biology. The mechanism of action for the anti-schistosomal drug PZQ remains poorly understood despite over forty years of research (reviewed in (Andrews et al., 1983; Day et al., 1992)). While Ca^2+^ signaling has been implicated in PZQ action since the earliest experiments on this drug (Chubb et al., 1978; Pax et al., 1978), PZQ's target has not yet been conclusively deorphanized. However, voltage-gated Ca^2+^ (Ca_v_) channels are likely central to the drug's molecular mechanism of action (Greenberg, 2005; Chan et al., 2013). Conceivably, PZQ could target a Ca_v_ channel directly (Kohn et al., 2001), or engage Ca_v_ channels indirectly via actions on other targets such as GPCRs or TRP channels (Babes et al., 2017; Chan et al., 2017; Gunaratne et al., 2018; Park et al., 2019).

**Fig. 1:**
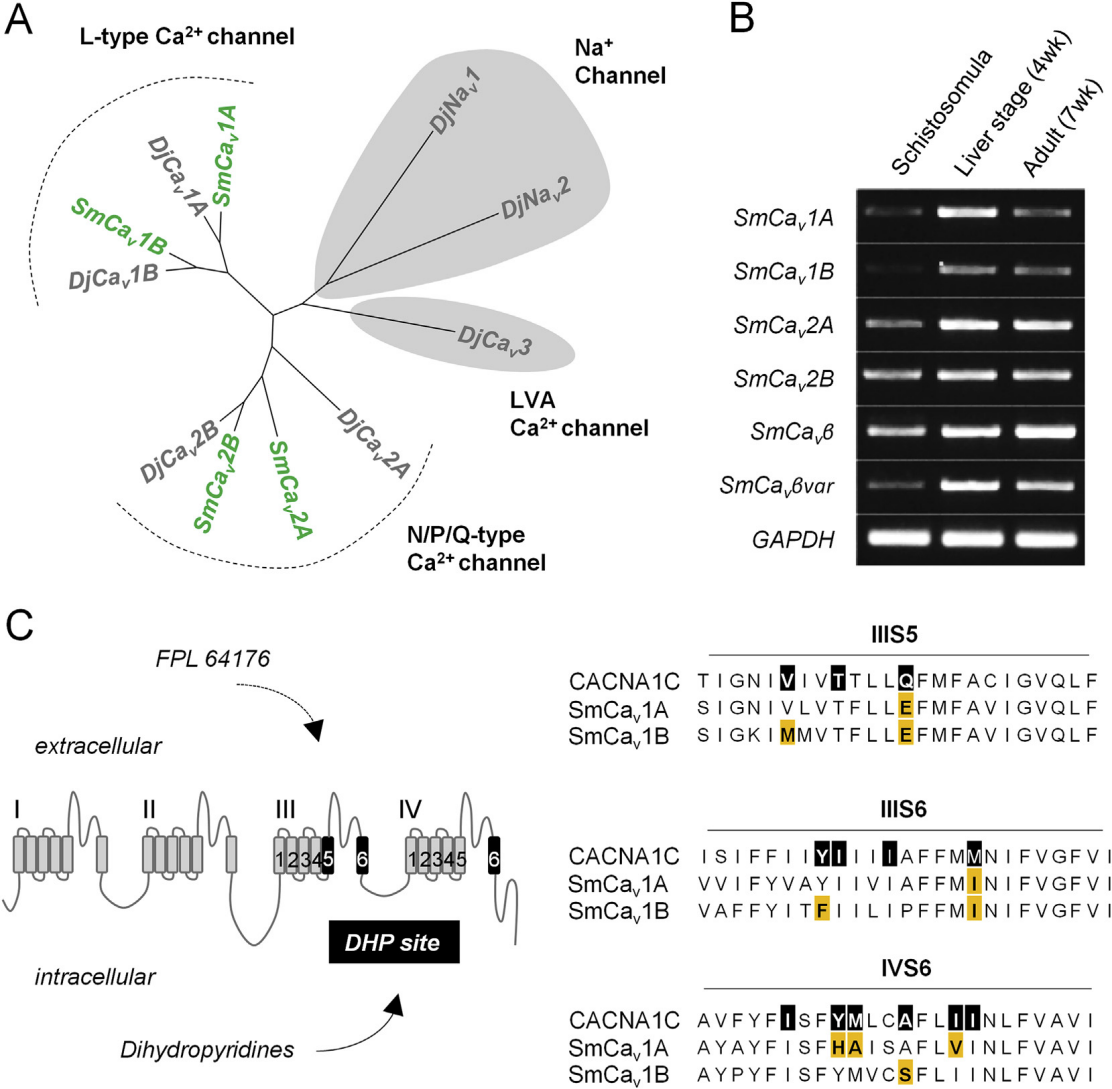
***Schistosoma mansoni* voltage-gated Ca**^**2+**^**channels. (A)** Cladogram of voltage-gated cation channels in free-living *Dugesia japonica* planarian flatworms (grey) and parasitic *S. mansoni* (green). **(B)** RT-PCR of Ca_v_α and accessory Ca_v_β subunits across the intra-mammalian life cycle stages, relative to the housekeeping gene GAPDH. **(C)** Schematic of dihydropyridine (DHP) Ca_v_ binding residues within the third and fourth transmembrane domains. Most interacting residues are within the transmembrane helices IIIS5, IIIS6 and IVS6. Black boxes = amino acids required for DHP binding and efficacy at mammalian Ca_v_s. Orange = amino acids not conserved in schistosome channels SmCa_v_1A (Smp_020270) and SmCa_v_1B (Smp_159990), relative to representative DHP-sensitive rat Ca_v_1.2 (NP_036649.2). (For interpretation of the references to color in this figure legend, the reader is referred to the Web version of this article.)

Regardless of whether Ca_v_ channels are anthelmintic receptors or downstream effectors of drug action, they are likely crucial for the activity of drugs that target worm neuromusculature given their role governing tissue excitability. Parasitic schistosomes possess a pared-down complement of voltage-gated cation channels relative to free-living flatworms (Fig. 1A), having lost voltage-gated sodium (Na_v_) channels and low voltage gated Ca^2+^ channels (Chan et al., 2016). This stripped-down repertoire of excitable channels may result in an increased dependence on Ca_v_s for voltage-sensitive cation influx. The *Schistosoma mansoni* genome encodes four high voltage gated Ca_v_ channels (Fig. 1A), two of which are predicted L-type Ca^2+^, or Ca_v_1, channels. Ca_v_1 channels mediate muscle excitability and contraction, and are present in the muscle fibers of free-living (Blair and Anderson, 1994; Cobbett and Day, 2003) and parasitic flatworms (Novozhilova et al., 2010). Contraction of isolated schistosome muscle fibers is Ca^2+^ dependent (Mendonca-Silva et al., 2006) and driven by inward Ca^2+^ currents that can be blocked by the Ca_v_ antagonist verapamil (Novozhilova et al., 2010). However, a limitation when studying drug targets in invertebrate organisms is the varied utility of chemical probes optimized for mammalian targets. The study of mammalian Ca_v_s has relied heavily on the use of small molecules such as the dihydropyridine (DHP), phenylalkylamine (PA) and benzothiazepine (BZ) classes of antagonists to probe channel function both in heterologous expression or tissue bioassays. However, these compounds exhibit a marked decrease in efficacy and potency when applied to schistosomes relative to mammalian channels - sometimes requiring high concentrations near the limit of compound solubility (Day et al., 1994; Novozhilova et al., 2010). This can confound interpretation of experimental data, especially considering that many of these same DHP mammalian Ca_v_ antagonists are also agonists at other Ca^2+^ channels at these higher concentrations. This may explain why the DHP nifedipine has anti-schistosomal effects on worms cultured *ex vivo* at the micromolar range (Silva-Moraes et al., 2013), since this compound also displays agonist activity at TRPA1 channels starting at high nanomolar concentrations (Fajardo et al., 2008).

Given the poor utility of DHP-based ligands as probes to study flatworm Ca_v_ channels, we sought to characterize the effects of the structurally distinct Ca_v_ agonist FPL-64176 on schistosomes. While FPL-64176 is a well characterized mammalian L-type Ca_v_ ligand, further breakthrough in heterologous expression of schistosome Ca_v_ channels will be required to demonstrate that this compound's effects on schistosomes are due to action on parasite channels. However, we have found that this ligand exerts profound effects on *Schistosoma mansoni* tissues *in vitro* and displays potential *in vivo* efficacy in a murine model of schistosomiasis.

## 2 Materials and methods

*Profiling Ca*_*v*_ *channel expression by RT-PCR.* Mixed sex *S. mansoni* RNA from various life cycle stages was extracted using TRIzol reagent (ThermoFisher Scientific cat. # 15596018) and the Purelink RNA Mini Kit with on column DNase treatment (ThermoFisher Scientific cat. # 12183025) and cDNA was synthesized using the High Capacity mRNA to cDNA kit (ThermoFisher Scientific cat. # 4387406). Primers shown in Supplemental Table 1 were designed against Ca_v_ and GAPDH sequences (Primer3Plus), and PCRs were performed using the FastStart Taq Polymerase kit (Millipore Sigma cat. # 4738357001). PCR product was run on a 1% agarose gel and cloned into pGEM-T Easy (Promega) for verification by sanger sequencing.

*Adult schistosome movement assays.* Female Swiss Webster mice infected with *S. mansoni* cercariae (NMRI strain) were sacrificed by CO_2_ euthanasia seven weeks after infection. Schistosomes were harvested by dissection of the mesenteric vasculature and cultured in DMEM (ThermoFisher cat. # 11995123) supplemented with HEPES (25 mM), 5% bovine calf serum (Sigma Aldrich cat. # 12133C) and penicillin-streptomycin (100 units/mL). Worms were cultured in 6 well dishes (4–5 worm pairs in 3 mL media per well) at either room temperature or 37 °C/5% CO_2_, depending in assays being performed. (S)-(−)-Bay K 8644 and FPL-64176 were purchased from Tocris Bioscience (cat. # 1403 & 1546, respectively). Praziquantel was purchased from Sigma Aldrich (cat. #P4668-1G). Compounds were solubilized in DMSO and added to culture media at concentrations indicated. Worm movement and morphology in response to drug exposure was recorded using a Zeiss Discovery v20 stereomicroscope and a QiCAM 12-bit cooled color CCD camera, controlled by Metamorph imaging software (version 7.8.1.0). Videos were acquired for 1 min at 4 frames per second, saved as a.TIFF stack, and imported into ImageJ for analysis. Maximum intensity projections for the.TIFF stacks that contained each frame of the 1-min recording were generated, and integrated pixel intensity values were measured for the resulting composite image to produce a quantification of total movement. Data = mean ± standard error for ≥3 experiments. All animal work was carried out with the oversight and approval of the Laboratory Animal Resources facility at the Medical College of Wisconsin and following ethical regulations approved by the Medical College of Wisconsin IACUC committee.

*Schistosome tegument surface protein biotinylation.* The surface of drug treated schistosomes was biotinylated following the method reported in (Braschi and Wilson, 2006) to image drug evoked changes to the tegument surface. Proteins on the surface of the parasite were biotinylated with EZ-Link sulfo–NHS–biotin (890 μM, ThermoFisher Scientific cat. # 21217) for 30 min at 4 °C, washed, labeled with FITC-streptavidin conjugate (Invitrogen cat. # SA1001) for 30 min at room temperature, and fixed in 4% paraformaldehyde (PFA). Worms were then mounted on an Olympus IX81 microscope and imaged using a Yogokawa spinning disk confocal (CSU-X-M1N) and an Andor iXon Ultra 888 EMCCD camera.

*Cell Proliferation Assay.* Cell proliferation was measured using the Click-iT Plus EdU Alexa Fluor 488 Imaging Kit (Invitrogen). Worms were dissected from the mesenteric vasculature of infected mice and incubated in culture media supplemented with test compound and 10 μM EdU overnight (ThermoFisher Scientific cat. #C10637). Worms were fixed in 4% PFA, permeabilized in PBS +0.3% Triton X-100 and incubated in detection solution, followed by DNA staining with Hoechst 33342.

*Transmission Electron Microscopy.* Adult worms were harvested and treated following the methods described in the movement assays and fixed in 2.5% glutaraldehyde/2% paraformaldehyde in 0.1 M sodium cacodylate (pH 7.3) at 4 °C overnight. Worms were washed (3 × 10 min) in 0.1 M sodium cacodylate and post-fixed in reduced 1% osmium tetraoxide (2 h on ice). Worms were washed (2 × 10 min) in distilled water and stained in alcoholic uranyl acetate (overnight, 4 °C). Worms were dehydrated in 50%, 75% and 95% MeOH, followed by successive rinses in 100% MeOH and acetonitrile (10 min each), and then incubated in a 1:1 mix of acetonitrile and epoxy resin (1 h) prior to 2 × 1 h incubations in epoxy resin. Worm sections were cut transversely, embedded overnight in epoxy (60 °C), and 70 nm ultra-thin sections were cut onto 200-mesh copper grids for staining in aqueous lead citrate for 1 min. Sections were imaged on a Hitachi H-600 electron microscope fitted with a Hamamatsu C4742-95 digital camera operating at an accelerating voltage of 75 kV.

*In Vivo Hepatic Shift Assay.* Mice harboring mature schistosome infections (6–7 weeks) were administered either FPL-64176 (8 mg/kg) or DMSO vehicle control by intraperitoneal injection. Doses and administration route were selected based on tolerated use of FPL-64176 in a mouse of transient dystonia (Jinnah et al., 2000). Mice were sacrificed by CO_2_ euthanasia 2 h after drug administration and worms were recovered from either the mesenteric vasculature, portal vein or liver. Data = mean ± standard error for 5 mice per cohort, with differences considered significant relative to DMSO control at p < 0.05 using the unpaired *t*-test.

*Comparative RNA-Seq.* Infected mice were treated with FPL-64176 as per the hepatic shift assay, except that three doses were given across successive days. Animals were sacrificed, worms were harvested from either the mesenteric vasculature (for the DMSO control treated cohort) or the liver (for the FPL-64176 treated cohort) and homogenized in TRIzol Reagent (Invitrogen). Total RNA was extracted and libraries were generated using the TruSeq Stranded mRNA kit (Illumina) and sequenced using the Illumina HiSeq 2500 system (high-output mode, 50 bp paired-end reads at 20 million reads per sample). Trimmed reads were mapped to the *Schistosoma mansoni* genome (v7.2) using HISAT2. EdgeR was used to identify differentially expressed genes (tagwise dispersion model, FDR adjusted p-value < 0.05) between FPL-64176 and DMSO control treated cohorts. FPL-64176 up-regulated transcripts were defined as those with an adjusted p value < 0.05 and a mean TPM ≥10 in the FPL-64176 cohort. FPL-64176 down-regulated transcripts were defined as those with an FDR-adjusted p value < 0.05 and a mean TPM ≥10 in the DMSO control cohort. Both lists were ranked by fold change and functional enrichment analysis was performed using the g:Profiler tool (Reimand et al., 2016). All RNA-Seq data has been deposited in the NCBI SRA database under SRA accession number PRJNA551487.

## 3 Results

### 3.1 The Ca_v_ agonist FPL-64176 damages schistosomes *in vitro*

Ca_v_ channels are intriguing anthelmintic targets given their role governing cellular excitability and their expression throughout the intra-mammalian life cycle stages (Fig. 1B). However, the study of these channels has been hampered by the fact that schistosomes are refractory to the DHP-class of ligands commonly used to study L-type Ca_v_ channel function (Day et al., 1994; Novozhilova et al., 2010). This is likely due to the fact that many amino acids that mediate DHP binding to mammalian Ca_v_ channels at transmembrane segments IIIS5, IIIS6 and IVS6 (Hockerman et al., 1997; Wang et al., 2018) are not conserved in schistosomes (Fig. 1C). In fact, schistosome Ca_v_s contain several amino acid substitutions that have been experimentally shown to confer a loss of DHP sensitivity in mammalian channels. For example, mutation of the Ca_v_1.2 Tyr 1179 position within helix IIIS6 (rat CACNA1C numbering) to Phe results in a loss of DHP binding affinity (Peterson et al., 1996), and SmCa_v_1B contains a Phe at this position. Mutation of the Ca_v_1.2 Gln 1070 position within helix IIIS5 to a Glu (Wappl et al., 2001) and the Met 1188 within helix IIIS6 to an Ile (Kwok et al., 2008) both result in a loss of DHP binding affinity, and both SmCa_v_1A and SmCa_v_1B contain a Glu and Ile at these respective positions.

**Fig. 2:**
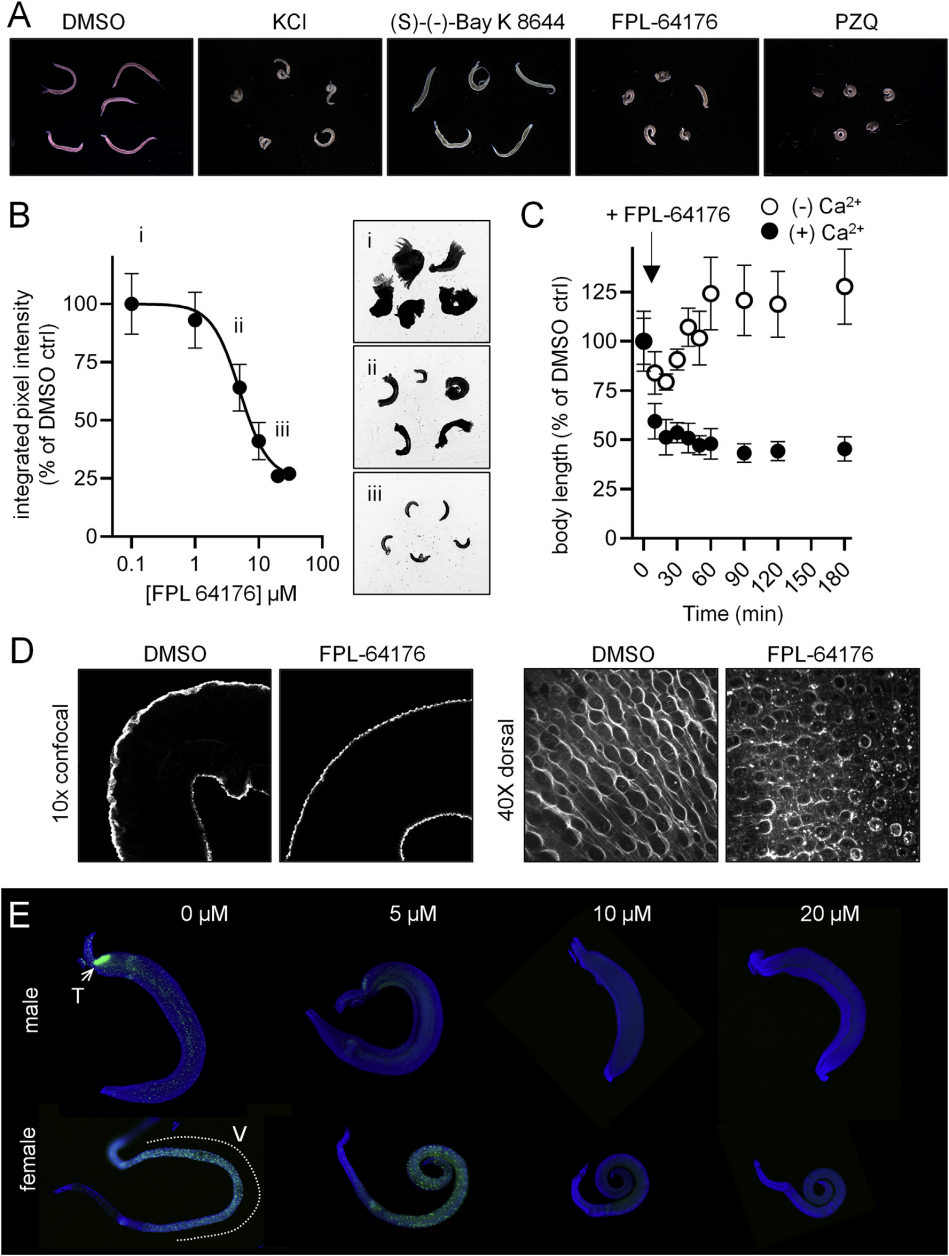
***In vitro* activity of FPL-64176 on cultured adult *S. mansoni*. (A)** Adult male schistosomes exposed to DMSO vehicle control, KCl (100 mM), (S)-(−)-Bay K 8644 (100 μM), FPL-64176 (20 μM) and PZQ (1 μM). **(B)** Dose-dependent effects of FPL-64176 (1 h incubation, room temperature) on schistosome movement. Left = quantified movement of male schistosomes. Right = composite images of 1 min recordings of worms exposed to (i) DMSO, (ii) FPL-64176 5 μM, (iii) FPL-64176 20 μM. **(C)** FPL-64176 (30 μM) evoked contraction of male schistosomes in normal DMEM (+Ca^2+^, solid symbols) and Ca^2+^-free DMEM (-Ca^2+^, open symbols). **(D)** Biotinylation of male schistosome tegument surface proteins. Worms were exposed to DMSO (left) or FPL-64176 (right). Left = confocal slice through the middle of the worm, revealing surface staining. Right = dorsal surface of worms. **(E)** Effect of FPL-64176 treatment (concentrations 0, 5, 10 and 20 μM) on EdU incorporation. After imaging movement in (B), worm culture media was supplemented with EdU (10  μM) and cultured a further 12 h at 37 °C/5% CO_2_. EdU incorporation (Green) is prominent in male testes (T) and female vitellaria (V), as well as somatic neoblasts distributed throughout the worm body. Blue = Hoechst DNA counterstain. (For interpretation of the references to color in this figure legend, the reader is referred to the Web version of this article.)

Lacking chemical probes, the schistosome voltage-sensitive channels have been crudely studied using high concentrations of potassium as a depolarizing stimulus. Worms cultured *in vitro* and exposed to KCl 100 mM rapidly contract (Fig. 2A), while the DHP ligand (S)-(−)-Bay K 8644 that is commonly used to experimentally activate mammalian L-type Ca_v_ channels evoked no obvious phenotypic changes even at concentrations as high as 100 μM. Therefore, we were interested in whether a structurally unrelated, non-DHP agonist may be an alternative tool to interrogate schistosome Ca_v_ signaling. The benzoyl pyrrole FPL -64176 is a full agonist at mammalian L-type Ca_v_ channels, as shown by bioassays on arterial preparations and electrophysiological measurement of L-type Ca_v_ currents (Zheng et al., 1991), but is structurally distinct from the DHP, PA or BZ classes of Ca_v_ ligands (Baxter et al., 1993) and does not bind Ca_v_ channels at any of the well-mapped binding sites for these three classes of compounds (Rampe and Lacerda, 1991; Zheng et al., 1991).

Schistosomes exposed to 10 μM FPL-64176 rapidly contract and exhibit a tonic paralysis reminiscent of PZQ exposure (Fig. 2A&B). FPL-64176 impaired schistosome movement with an IC_50_ of 5.0 ± 1.0 μM (Fig. 2B), in line with the active concentrations used in mammalian bioassays (Zheng et al., 1991). Worm contraction was Ca^2+^ dependent, consistent with the hypothesis that the effects of FPL-64176 are mediated by Ca_v_ channels, since worms exposed to FPL-64176 did not display sustained contractions when cultured in Ca^2+^ free-media (Fig. 2C).

Given the proposed utility of schistosome Ca_v_ channels as anthelmintic targets and the similarity between FPL-64176 and PZQ-evoked phenotypes, we were interested in assessing whether FPL-64176 exposure may have potential anti-parasitic effects. We assessed drug-evoked alteration of the parasite tegument surface, a common hallmark of anthelmintics such as PZQ (Mehlhorn et al., 1981). After performing motility assays (Fig. 2A&B), worms were cultured overnight (37 °C/5% CO_2_) in the continued presence of FPL-64176 and then processed to biotinylate tegument membrane proteins. The biotinylated surface of the worm was then stained with FITC-streptavidin to visualize the tegument apical membrane by confocal microscopy (Fig. 2D). The *S. mansoni* tegument surface normally contains an ordered, regular distribution of tubercles. However, the tegument surface of FPL-64176 treated worms was disrupted, displaying an irregular tubercle distribution and ‘blebbing’ or blistering of the surface (Fig. 2D&E).

In addition to assessing damage to the parasite surface, we also measured drug-evoked changes to parasite mitotic activity. After worm motility had been assessed, the thymidine analog EdU (10 μM) was added to drug containing culture media and samples were harvested after a further 12 h in culture (37 °C/5% CO_2_). The reproductive tissues of the male (testes) and female (ovaries, vitellaria) worms are highly proliferative, and parasites also possess abundant mitotically active pluripotent stem cells distributed that are throughout the worm body (Collins et al., 2013). FPL-64176 treatment inhibited cell division in a dose-dependent manner, completely blocking mitosis of somatic neoblasts and both male and female germ line tissues at 10 μM (Fig. 2E).

### 3.2 The effects of FPL-64176 are temperature dependent

**Fig. 3:**
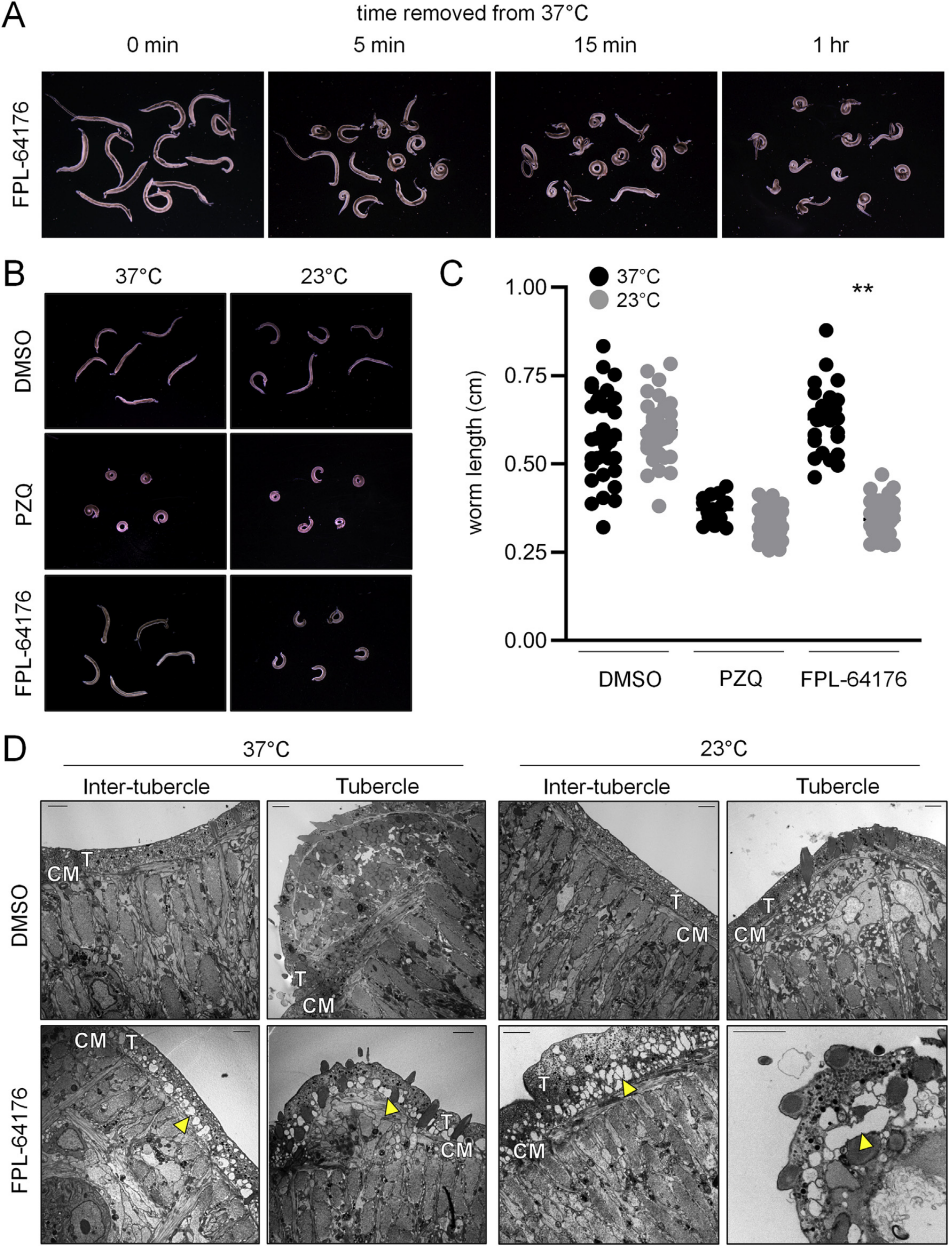
**FPL-64176 evoked contraction is temperature-dependent. (A)** Worms exposed to FPL-64176 (20 μM) for 12 h at 37 °C and imaged at 37 °C (left), and 5 min, 15 min and 1 h after being placed at 23 °C (right). **(B)** Comparison of DMSO, FPL-64176 (20 μM) and PZQ (1 μM) effects on male schistosomes at 37 °C and 23 °C. **(C)** Quantification of contraction, measured as worm body length (centimeters), for treatments shown in (B). Symbols = individual worm measurements. Black symbols = 37 °C. Grey symbols = 23 °C. **(D)** Transmission electron microcopy images of schistosome transverse cross sections showing the effect of FPL-64176 (20 μM) on tissue ultrastructure at 37 °C and 23 °C. Scale bar = 2  μm. T = tegument syncytium. CM = circular muscle layer. Yellow arrow = drug-evoked vacuoles distributed at the basal tegument. (For interpretation of the references to color in this figure legend, the reader is referred to the Web version of this article.)

While conducting these *in vitro* assays, we observed an interesting temperature-dependent effect of FPL-64176. Our initial movement assays were performed at room temperature (23 °C). This is common for schistosome *in vitro* drug screening, since worms display no overt changes in morphology or viability when cultured at room temperature for the time scale (~1 h) required for these acute assays (Fetterer et al., 1981). We observed that FPL-64176 was much less effective on worms treated at 37 °C, where higher concentrations of drug (40–50 μM) were required to cause contractile paralysis, even after treatments as long as 24 h. However, when apparently unaffected worms treated with FPL-64176 (20 μM) at 37 °C were placed on a lab bench at room temperature, they rapidly acquired the contractile phenotype previously observed (Fig. 3A&B). DMSO vehicle control treated worms were unaffected by temperature change, displaying similar morphology at both 37 °C (length = 5.9 ± 1.0 mm) and 23 °C (length = 6.0 ± 0.8 mm), and PZQ (1 μM) evoked shrunken, contractile paralysis regardless of temperature (37 °C length = 3.7 ± 0.4 mm and 23 °C length = 3.5 ± 0.5 mm). FPL-64176 (20 μM) treated worms were uncontracted at 37 °C (length = 6.3 ± 1.0 mm), appearing morphologically similar to DMSO controls, but at 23 °C they displayed the same contractile phenotype as PZQ (length = 3.2 ± 0.4 mm) (Fig. 3C).

**Supplementary Fig. 1:**
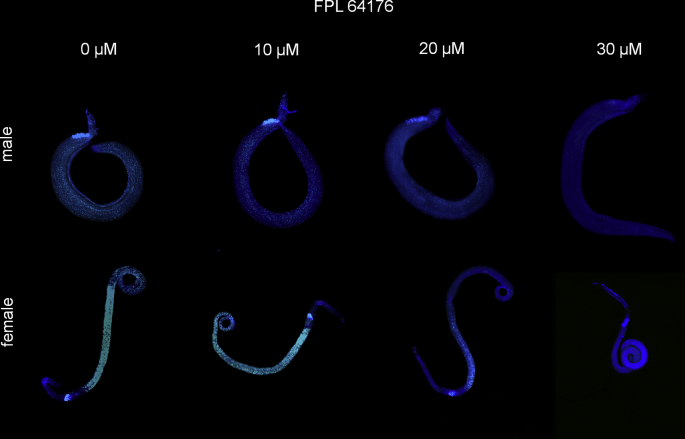
**Effect of FPL-64176 on schistosome mitotic activity at 37°C.** Worms treated with FPL-64176 (0, 10, 20 and 30 μM) and EdU (10 μM) at 37 °C / 5% CO_2_ for 12 h. EdU incorporation = Green. Hoechst DNA counterstain = Blue.

The contractile effects of FPL-64176 exhibited a clear temperature dependence. Therefore, we assessed whether drug-evoked changes in tegument integrity and tissue ultrastructure were also temperature dependent. The schistosome tegument sits above orthogonal layers of circular and longitudinal body wall muscle, but the interactions between these two tissues are not well defined. Not all experimental manipulations that cause muscle contraction damage tegument, and vice-versa (Bricker et al., 1983). Adult male worms treated with FPL-64176 (20 μM) at both 37 °C (uncontracted) or room temperature (contracted), as well as DMSO vehicle control, were fixed and imaged by transmission electron microscopy (TEM) to examine ultrastructural differences. DMSO control worms cultured at either 37 °C or 23 °C displayed no obvious defects. On the other hand, FPL-64176 treatment resulted in a dramatic accumulation of vacuoles within the tegument syncytium, just above the basement membrane (Fig. 3D). These vacuoles were pervasive, appearing across the entire surface of FPL-64176 treated samples regardless of whether worms were cultured at 37 °C or 23 °C. FPL-64176 exerted effects on worm mitotic activity at both 37 °C and 23 °C, but was slightly more potent at 23 °C (comparison of Fig. 2E at 23 °C with Supplemental Fig. 1 at 37 °C).

### 3.3 FPL-64176 is active against parasites *in vivo*

**Fig. 4:**
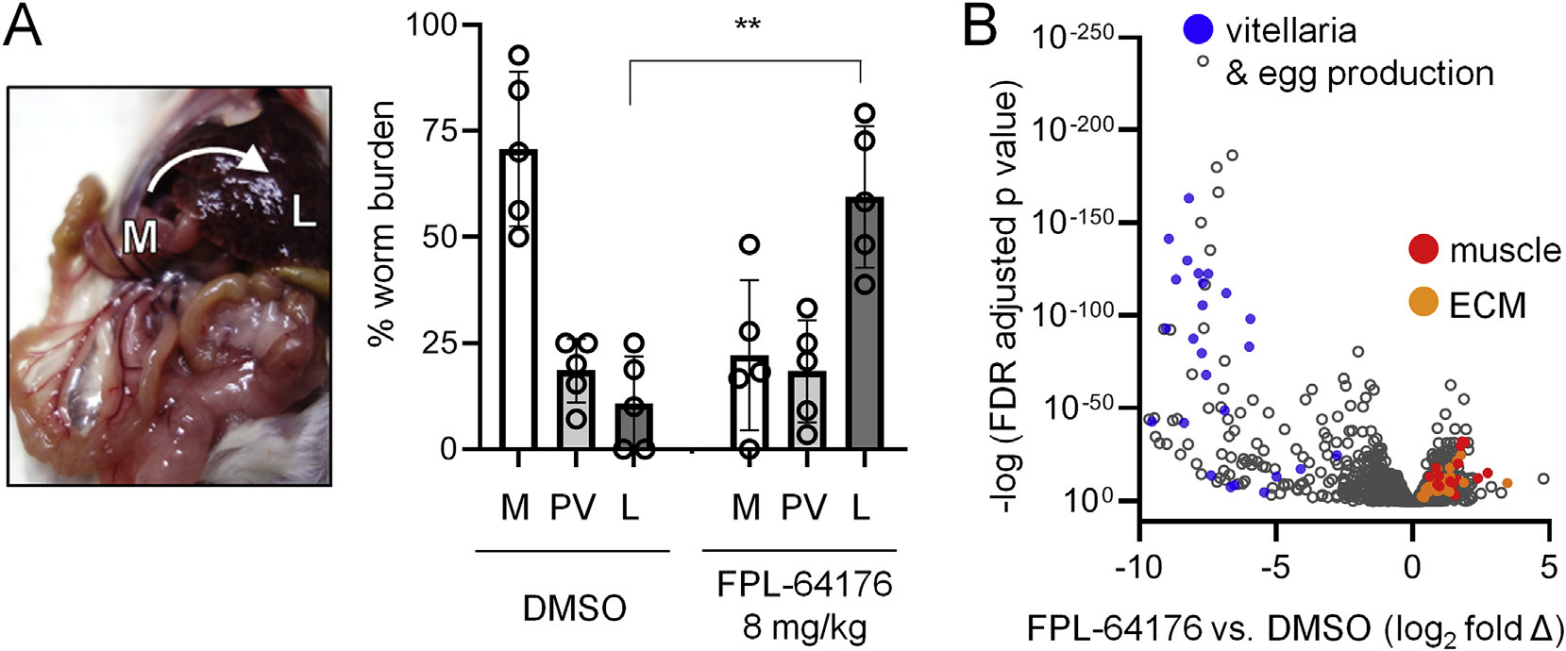
***In vivo* effects of FPL-64176 in a murine model of schistosomiasis. (A)** Hepatic-shift of worms following DMSO or FPL-64176 (8 mg/kg) administration. Left = Mature worms normally reside within the mesenteric vasculature around the intestines. Following anthelmintic treatment, worms shift along the haptic portal circulation and are lodged in the liver. Right = Quantification of worm burden in the mesenteries (M), portal vein (PV) and liver (L) 2 h after drug treatment. **p < 0.001. **(B)** Volcano plot of differentially expressed transcripts in worms harvested from mice following three days FPL-64176 treatment. Blue = transcripts annotated as involved in egg production or known vitellaria markers. Red = transcripts with muscle GO term annotations. Orange = transcripts with ECM GO term annotations (see Table 1). (For interpretation of the references to color in this figure legend, the reader is referred to the Web version of this article.)

Given that FPL-64176 treated worms closely resemble worms treated with PZQ *in vitro* (tonic contractile paralysis, tegument damage), we assessed whether FPL-64176 possessed antiparasitic activity against schistosomes *in vivo*. Mice harboring mature *S. mansoni* infections were administered either DMSO vehicle control or FPL-64176 (8 mg/kg) by IP-injection and sacrificed 2 h later to determine whether drug treatment caused a shift of worms from the mesenteric vasculature, where mature parasites reside during normal infection, to the liver, where worms are eliminated following chemotherapy (Mehlhorn et al., 1981). While just 13 ± 4% of worms were found in the livers of control mice, 58 ± 6% of worms in FPL-64176 treated mice had shifted to the liver (p value < 0.001, Fig. 4A). The dose of FPL-64176 was not increased further due to the potential for host toxicity. Instead, studies on longer term changes to schistosome biology were performed, harvesting worms after daily administration of compound for 3 consecutive days. Schistosomes were harvested from mouse mesenteries (DMSO control treated mice) or livers (FPL-64176 treated mice) and comparative RNA-Seq was performed to assess changes in gene expression (Fig. 4B). GO-term and KEGG pathway analysis of differentially expressed gene products revealed enrichment of terms associated with muscle function (e.g. myosin complex, contractile fiber part, myofilament, troponin complex), cell-cell adhesion or ECM-mediated signaling (e.g. ECM-receptor interaction, ECM structural constituent, ECM-receptor interaction, integrin-mediated signaling) (Table 1 and Fig. 4B, red). Many down regulated gene products reflected changes in female reproductive biology (Fig. 4B, blue), including known vitellaria markers such as tyrosinase and SOD (Wang and Collins, 2016) and various transcripts annotated as eggshell proteins or eggshell synthesis domain containing proteins. These changes are consistent with FPL-64176 inhibition of mitosis in the female vitellaria (Fig. 2E).

**Table 1:** **Functional annotation of transcripts up-regulated with FPL-64176 treatment.** Differentially expressed gene products were functionally annotated using g:Profiler, revealing an enrichment of transcripts associated with muscle and ECM function.

Term name	Source	Adj. p val.
ECM-receptor interaction	KEGG	4.15E-07
ECM structural constituent	GO:MF	3.77E-06
integrin-mediated signaling	GO:BP	3.24E-05
cell-cell adhesion	GO:BP	8.01E-03
collagen trimer	GO:CC	6.08E-03
myosin complex	GO:CC	8.03E-04
contractile fiber part	GO:CC	2.16E-02
striated muscle thin filament	GO:CC	2.16E-02
contractile fiber	GO:CC	2.16E-02
myofilament	GO:CC	2.16E-02
troponin complex	GO:CC	2.16E-02
sarcomere	GO:CC	2.16E-02
myofibril	GO:CC	2.16E-02

## 4 Discussion

Schistosome Ca_v_ channels have been an area of interest for decades, given their role mediating parasite muscle and tegument excitability and involvement in the mechanisms of action for various anti-schistosomal drugs (Pax et al., 1978). However, the study of flatworm Ca_v_ channels has been hampered by several technical difficulties. Heterologous expression has proved difficult (no flatworm Ca_v_1 has been successfully functionally expressed to date). Recording endogenous Ca_v_ currents from isolated schistosome muscle cells is technically challenging - initial attempts to resolve these currents were unsuccessful (Day et al., 1993). While current recordings were eventually achieved, they have peak amplitudes on the scale of tens of pA and rapidly decline within several minutes (Novozhilova et al., 2010). Therefore, many studies of schistosome Ca^2+^ signaling have relied on pharmacological tools, as imperfect as these are for studying invertebrate channels. The commonly used DHP class of antagonists display varying efficacy and poor potency on schistosomes. Given the lack of small molecule agonists, experimental activation of schistosome Ca_v_ channels has typically relied on supplementing culture media with high concentrations of potassium (~60–100 mM) as a depolarizing stimulus (Day et al., 1994). Therefore, there is a need to identify putative schistosome Ca_v_ channel agonists to study parasite Ca^2+^ signaling and muscle physiology.

### 4.1 The non-DHP Cav agonist FPL-64176 is active against schistosomes

A likely explanation for the inactivity of DHP ligands on Ca_v_ channels is a lack of conservation of amino acid residues in the Ca_v_ channel DHP-binding site (Fig. 1C). DHP insensitivity has been reported for several other invertebrate Ca_v_1 channels. Chimera experiments with the L-type Ca_v_ cloned from the housefly *Musca domestica* revealed an insensitivity to DHP agonists (Sinnegger et al., 1997), and L-type Ca_v_s from the jellyfish *Cyanea capillata* (Jeziorski et al., 1998), the sea squirt *Halocynthia roretzi* (Yamaguchi et al., 2000) and the snail *Lymnaea stagnalis* (Senatore et al., 2011) all exhibit reduced sensitivity to DHP antagonists. Given that the small molecule FPL-64176 is structurally distinct from the DHPs and has a different Ca_v_ binding site, we reasoned this compound may be an effective tool to probe flatworm Ca_v_ function. Successful heterologous expression of SmCa_v_1A or SmCa_v_1B will be needed for definitive validation of FPL-64176 as a bonda-fide schistosome L-type Ca_v_ ligand. However, the drug exhibits pronounced, Ca^2+^ dependent effects on cultured *S. mansoni*. This phenotype was curious in that it appears to be temperature-dependent. This may be due to slower Ca_v_ channel inactivation kinetics at 23 °C than 37 °C, since mammalian Ca_v_1.2 and Ca_v_1.4 display a 10–50 fold slower inactivation at room temperature (Peloquin et al., 2008). Alternatively, this effect could be due to altered muscle excitation-contraction (E-C) coupling. Relatively little is known about schistosome muscle physiology, but the body-wall muscle appears to be analogous to mammalian smooth muscle (Sulbaran et al., 2015) and there is a long-standing literature in mammalian bioassays showing that cooler temperatures increase muscle tension, in part via temperature-dependent activity of myosin light chain kinase and phosphatase (Nasu, 1990; Mitsui et al., 1994). It is also entirely possible that temperature sensitive TRP channels, such as schistosome TRPM homologs (Bais and Greenberg, 2016), are activated by cool temperatures and influence muscle tone.

### 4.2 FPL 64176 disrupts tegument adjacent the basement membrane

Regardless of temperature, FPL-64176 causes extensive vacuolization of the basal membrane of the tegument syncytium (Fig. 3D). Tegument damage is a common feature of anthelmintics, which cause ‘blebbing’ at the apical membrane on the surface of the worm and degeneration of the tegument tubercles. The tegument is a complex structure, with cell bodies located within the worm parenchyma, connected to the syncytial surface of the worm by projections that traverse the worm body wall. Maintaining this dynamic structure is likely an energetically demanding task, given that the worm surface rapidly turns over with a half-life of ~6 h (Roberts et al., 1983). This requires the trafficking of proteins and membrane precursors from the cell bodies, along the cytoskeleton and towards the surface of the worm (Zhou and Podesta, 1989). FPL-64176 may influence the tegument by disrupting the thin layer of circular muscle that is in contact with the basement membrane of the tegument syncytium, or via Ca^2+^ evoked changes to the cytoskeleton to impair vesicle trafficking. This could be through action on targets at either the tegument or muscle, since these adjacent tissues are electrically coupled (Thompson et al., 1982). In related planarian flatworms, body wall muscle functions as connective tissue, secreting matrisome proteins (Cote et al., 2019). Many of these gene products are differentially expressed following FPL-64176 treatment (Fig. 4B), consistent with *in vitro* drug effects on muscle cells and the tegument near the basement membrane (Fig. 3D).

### 4.3 Relevance to PZQ mechanism of action

While PZQ's molecular mechanism of action remains poorly understood, its effects are closely associated with Ca^2+^ signaling, and Ca_v_1 channels in particular (reviewed in (Chan et al., 2013)). Therefore, it is worth noting which effects of PZQ are recapitulated by FPL-64176. Both compounds evoke identical, Ca^2+^-dependent tonic contractile paralysis as well as damage to the worm tegument (Xiao et al., 1984) (Fig. 2). Both compounds evoke a parasite hepatic shift *in vivo* (Fig. 4A). However, unlike FPL-64176, PZQ's affects are not temperature dependent (Fig. 3B). One possible explanation for this is that PZQ does not act on L-type Ca_v_s directly, but instead acts on a different Ca^2+^-channel such as a schistosome TRPM channel (Park et al., 2019). The contractile tension of schistosome musculature is temperature dependent (Fetterer et al., 1978), and PZQ has recently been shown to activate a thermosensitive human TRPM8 (Babes et al., 2017; Gunaratne et al., 2018). This does not preclude a role for L-type Ca_v_s downstream of a putative PZQ interaction with a TRP channel, but may explain why the effects of FPL-64176 are similar but not identical to PZQ.

This report is the first study of a putative small molecule agonist of schistosome Ca_v_ channels, identifying FPL-64176 as a promising pharmacological tool to investigate schistosome Ca^2+^ signaling and the role of these pathways in the physiology of excitable cells such as the parasite tegument and muscle. Additionally, while FPL-64176 is likely not a viable anthelmintic due to action on host Ca_v_ channels, the structure of this molecule may be a useful starting point for derivatization and the design of ligands with increased parasite-selectivity.

## Declarations of interest

None.

## Appendix A Supplementary data

Supplementary Table 1 List of Primers used to amplify SmCa_V_ and SmGAPDH transcripts. Supplementary Table 1
