# Supplementary material for: Anti-schistosomal action of the calcium channel agonist FPL-64176
Source: Int J Parasitol Drugs Drug Resist. 2019 Sep 14;11:30–8. doi: 10.1016/j.ijpddr.2019.08.006 (PMC6796685; doi:10.1016/j.ijpddr.2019.08.006)
Supplement: Supplementary Table 1 — List of Primers used to amplify SmCaV and SmGAPDH transcripts. [file mmc1.docx]

**Supplementary Table 1**

| **Smp ID** | **Gene Name** | **Forward Primer** | **Reverse Primer** |
| --- | --- | --- | --- |
| Smp_004730 | Ca_V_ 1A | GTTGCATTGGGTTTTGCTTT | TTCCGTCCAACCTTCCATAG |
| Smp_340320 | Ca_V_ 1B | AACCGGTGGATACCCATACA | CAGCTATGGCACGTTTCTCA |
| Smp_020270 | Ca_V_ 2A | CTTGCTGCCCTTAAAGAACG | TCAAGGCCAACAATTGCATA |
| Smp_159990 | Ca_V_ 2B | TCCGATACGGAAATGAAAGC | GCACGAGGTCCATTGTATCC |
| Smp_141660 | Ca_V_ β | AGGAAAATGCTCGTCAGGAA | CAACATCGTATGGAGGCAAA |
| Smp_135140 | Ca_V_ β var | TGCTGGTAGCGTTGGTAGTG | TTCACGTTGCTTTGCTTGAC |
| Smp_056970 | GAPDH | TAAGGACGGGGCTGAATATG | TGACCTTAGCCAGAGGTGCT |
